# A clinical study on plasma biomarkers for deciding the use of adjuvant corticosteroid therapy in bronchopulmonary dysplasia of premature infants

**DOI:** 10.7150/ijms.58650

**Published:** 2021-04-29

**Authors:** Haiyan Zhu, Yian Tian, Huaiping Cheng, Yafei Zheng, Wei Wang, Tianping Bao, Rong Wu, Zhaofang Tian

**Affiliations:** 1 Department of Neonatology, The Affiliated Huaian No.1 People's Hospital of Nanjing Medical University, Huai'an, Jiangsu, China.; 2 University of Barcelona, TPM-DTI, Barcelona, Catalunya, Spain; 3 Neonatal Medical Center, Huai'an Maternity and Child Healthcare Hospital, Yangzhou University Medical College, Huai'an, Jiangsu, China.

**Keywords:** bronchopulmonary dysplasia, proteome, biomarker, corticosteroids

## Abstract

**Objective:** The study was designed to investigate some plasma markers which help us to decide the use of adjuvant corticosteroid therapy in bronchopulmonary dysplasia (BPD) of premature infants.

**Methods:** Thirty BPD infants were treated by dexamethasone. Among these cases, dexamethasone was significant effective in 10 cases, and no significant effective in 20 cases. These patients were divided into two groups as the significant effect (SE) group (n=10) and the non-significant effect (NE) group (n=20) according to the curative effect of dexamethasone. Fifteen non-BPD infants with gestational age and gender matching were selected as the control group. Plasma samples before and after dexamethasone treatment were collected from three infants chosen randomly from SEG for the data-independent acquisition (DIA) analysis. ELISA was further used to detect the levels of differential proteins LRP1 and S100A8 in all individuals, including SE, NE and control groups.

**Results:** DIA analysis results showed that after dexamethasone treatment, there were a total of 52 plasma proteins that showed significant differences, of which 43 proteins were down-regulated and 9 proteins were up-regulated. LRP1 and S100A8 were two plasma proteins that were significantly changed after dexamethasone treatment. Compared with the control group, plasma LRP1 was significantly increased in BPD. Interestingly, the plasma concentration of LRP1 in the NE group was significantly higher than that in the SE group. S100A8, as an indicator of plasma inflammation, was significantly higher in BPD than the control group. Unlike LRP1, there was no significantly difference between the SE and NE group (P=0.279) before dexamethasone treatment.

**Conclusion:** Elevated plasma LRP1 and S100A8 in BPD infants are two indicators that correlated with the efficacy of dexamethasone, and might be used as biomarkers for deciding the use of adjuvant corticosteroids therapy in the BPD.

## Introduction

Bronchopulmonary dysplasia (BPD) is the result of a complex process in which several prenatal and/or postnatal factors interfere with lower respiratory tract development, leading to a severe, lifelong disease. In the underlined disease the lungs and bronchi are damaged which destroys tissues in the alveoli of the lungs. Although its definition, epidemiology, pathophysiology, prevention, and management have continued to evolve. The prematurity of newborns is the major risk factor for BPD. However, the severity of the BPD differs from infant to infant 1. Each year, over 10000 new BPD cases are reported in the US 2. In China, the incidence of BPD in ultra-premature or ultra-low birth weight infants (ELBW) varied in 12.5%-48.1% 3,4.

Many non-pharmacologic and pharmacologic therapies have been suggested for lowering the lung's injury and tend to enhance the recovery of patients 5 *i.e.,* protective ventilation strategies, surfactant supplementation, optimal oxygen saturation goals, and the antenatal corticosteroids 6. The presently used drug therapies for BPD treatment are as following, caffeine 7, bronchodilator 8, surfactant 9, diuretics 10, inositol 11, corticosteroids^12^, viral immunization 13 and cardiac medications 14 etc. However, these treatments are partially effective; the majority of the interventions tried so far have not proven to be beneficial in rigorous meta-analyses of eligible studies. And still, there is a lack of significant therapy for curing the underlined disease.

In BPD the corticosteroids are used to reduce the inflammatory processes that lead to the pathogenesis of the disease. Corticosteroids have a highly potent inhibitory activity against inflammation and have an important contribution to the prevention of BPD. With the risks for Perforated Bowel, hypertrophic cardiomyopathy, major neurosensory disability, and cerebral palsy (CP), the “early” dexamethasone therapy has not been recommended, and the “later” dexamethasone therapy can be used selectively 15. The statement policy of an American Academy of Pediatrics was published in 2010 regarding the use of postnatal corticosteroids and admitted that the available data were not enough to support the corticosteroids recommendations 16. Since the BPD infants have different therapeutic response to dexamethasone therapy, so it has an important clinical value to find biomarkers that can evaluate the effectiveness of the BPD affected infants for dexamethasone therapy.

DIA is a non-standard quantitative technique employed for the identification of differentially expressed proteins. This technique is well suited for the evaluation of lung injury in children to identify key biomarkers 17. The purpose of the study is to find differentially expressed plasma proteins of BPD infants responsive to dexamethasone therapy by DIA technique to promote effective treatment of BPD infants.

## Materials and Methods

### Patients

The existing work was conducted in the Neonatal Intensive Care Unit of The Affiliated Huaian No.1 People's Hospital of Nanjing Medical University. At 36 weeks of postmenstrual age from Jan 2019 to Mar 2020, premature babies with gestational period < 32 weeks were detected with BPD. The diagnosed was based on “New consensus on BPD definitions and diagnostic criteria, developed in 2018 NICHD Seminar” (abbreviated as 2018 NICHD) 18. The following inclusion criterion is applicable in the subjects: clinical diagnosis as BPD grade Ⅲ, the details were as follows. Treatment with more than 21% oxygen for minimum of 28 days, required for 30% oxygen and/or positive pressure or discharge from the hospital, whichever comes first. Any one of the following exclusion criteria was applicable in the current study: I) There existed some congenital malformations such as digestive tract, respiratory tract, central nervous system, and heart; II) There existed ventilator-associated pneumonia (VAP); III) any glucocorticoid e.g., dexamethasone, hydrocortisone, budesonide, etc. administrated before the research. IV) Clinical signs of infection were existed. Totally, thirty BPD infants were enrolled in this study.

All eligible cases were treated with a 10-day dexamethasone course after diagnosing with BPD. The details are as follows: Infants received a 10-day tapering course of dexamethasone sodium phosphate (0.15 mg/kg per day for 3 days, 0.10 mg/kg per day for 3 days, 0.05 mg/kg per day for 2days, and 0.02 mg/kg per day for 2 days; total of 0.89 mg/kg over 10 days 19. Plasma samples were retained before and after dexamethasone therapy, respectively. The curative effect was judged immediately after 10-day course of dexamethasone. According to the curative effect of dexamethasone, these BPD infants were divided into two groups as the significant effect (SE) group (n = 10) and the non-significant effect (NE) group (n = 20). SE was defined as patients with no need of respiratory support and oxygen dependence. NE was defined as lowering the inhaled oxygen concentration and/or the decrease in positive pressure support, or no change in inhaled oxygen concentration and/or no improvement in respiratory support. In addition, 15 non-BPD infants with gestational age and gender matching were selected as the control group. The approval for this clinical research was provided by the ethics committee of The Affiliated Huaian No.1 People's Hospital of Nanjing Medical University, and the relevant informed consent was signed from the parents before the trial. The registration number of the Chinese Clinical Trail Registry is ChiCTR2000033796 (http://www.chictr.org.cn/historyversionpub.aspx?regno=ChiCTR200003796).

### DA analysis

Plasma samples before and after dexamethasone treatment were collected from three infants chosen randomly from SEG for the data-independent acquisition (DIA) analysis (Guangzhou Jidio Biotechnology Co. Ltd, Guangzou, China). Plasma samples were transferred into lysis buffer, and the proteins in lysates were concentrated, followed by proteins digesting via sequence-grade modified trypsin using the method of BCA Protein Assay Kit (Guangzhou Jidio Biotechnology Co. Ltd, Guangzhou, China). The digested peptides were dried to prepare for the analysis of nano-HPLC-MS/MS. Unprocessed data obtained from DIA were analyzed and sort out via Spectronaut X (Biognosys AG, Switzerland) with default parameters. After Student's t-test, various expressed proteins (having Q value < 0.05 and Absolute AVG log2 ratio > 0.58) were filtered.

### Enzyme-linked immunosorbet assay (ELISA)

To confirm the differentially expressed proteins identified by DIA, we focused on LDL receptor-related protein 1 (LRP1) and S100 calcium-binding protein A8 (S100A8). Venous blood was centrifuged at 1,000 × g to obtain a supernatant. The levels of LRP1 and S100A8 in the supernatant were identified via the ELISA kit (abcam, Cambridge, England) for the control group and the BPD group (including pre-and post- dexamethasone administration), following the given instructions of the manufacturer.

### Statistical analysis

GraphPad Prism 8.0 (GraphPad Software Inc.) was used for statistical processing. The difference was evaluated by paired Student t test (2-group comparisons) or ANOVA followed by the post hoc Tukey's Multiple Comparison Test (multi-group comparisons) as appropriate. Data are presented as the mean ± SD. P < 0.05 was considered statistically significant.

## Results

### The characteristics of BPD infants

A total of 30 eligible infants were included in this study, of which 10 cases showed significant effect (SE), while 20 non-significant effects (NE) after dexamethasone treatment. The two groups' patients had similar perinatal characteristics, as depicted in Table 1. The difference was that three patients in the NE group had ventilator associated pneumonia, while none in the SE group. Three BPD patients were randomly selected from the SE group. The perinatal characteristics were presented in Table 2. The perinatal characteristics of the selected three BPD patients for DIA analyses were representative.

### Differentially expressed proteins before and after dexamethasone administration in SE group

**Figure 1:**
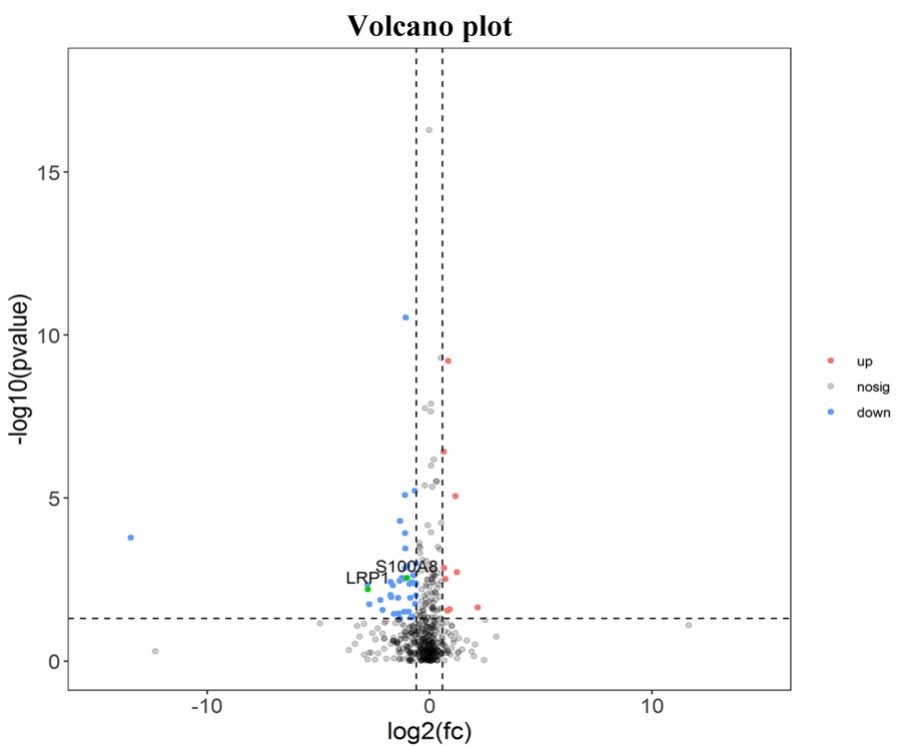
Distinct pulmonary proteomic patterns in T1 group and T2 group. Red represents up-regulated protein, blue represents down regulated protein, and black has no difference (T1 Pre-dexamethasone administration; T2 Post-dexamethasone administration).

**Figure 2:**
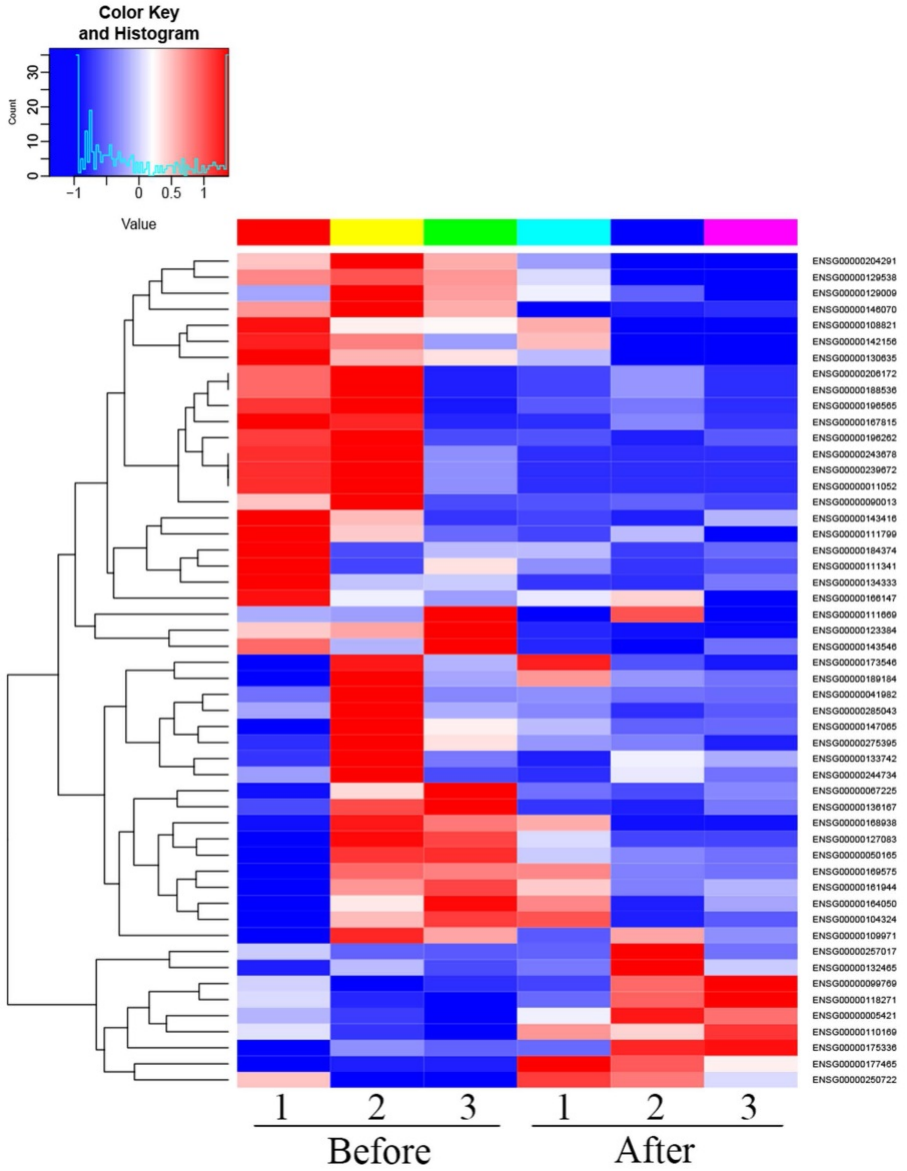
Heat map of differentially expressed proteins (T1 Pre- dexamethasone administration; T2 Pos-t dexamethasone administration).

There were 52 proteins displayed significant expression changes before and after dexamethasone treatment. In this cohort, the upregulation of 9 proteins and downregulation of 43 proteins were found, as indicated in Table 3. The volcanic map directly showed the location of various proteins between the groups that were compared, as depicted in Fig. 1. Hierarchical cluster analysis and Z-score homogenization were used to map the differentially expressed proteins in groups that were compared (each column represents a sample and each row represents a protein). The protein expression in different samples is expressed in different colors. The red color represents the elevated expression, while the blue color showed decreased expression level, as depicted in Fig. 2.

### Bioinformatics analyses of the differentially expressed proteins

The analysis of GO (The Gene Ontology) indicated that the differentially expressed proteins participated in the cofactor metabolic process (GO : 0051186) and the catabolic process (GO : 0009056). The most significantly affected molecular functions were catalytic activity, binding, and structural activity relationship (Supplementary Online Fig. S1). Pathway analysis by KEGG suggested that the most significantly affected cascades were purine metabolism, pyrimidine metabolism, drug metabolism-other enzymes (Supplementary Online Fig. S2). We further map genes to each term of the DO database (http://disease-ontology.org/). The results of DO enrichment were shown in Supplementary Online Fig. S3. The DO items that pulmonary hypertension and persistent fetal circulation syndrome might relate to the pathogenesis of BPD. Then, we map the gene to each term of the react database (https://react.org/). The underlined results were depicted in Supplementary Online Fig. S4, and the top twelve Reactome items were significantly enriched in the gene (p < 0.05).

### Validation of changed LRP1 and S100A8levels in the plasma samples

**Figure 3:**
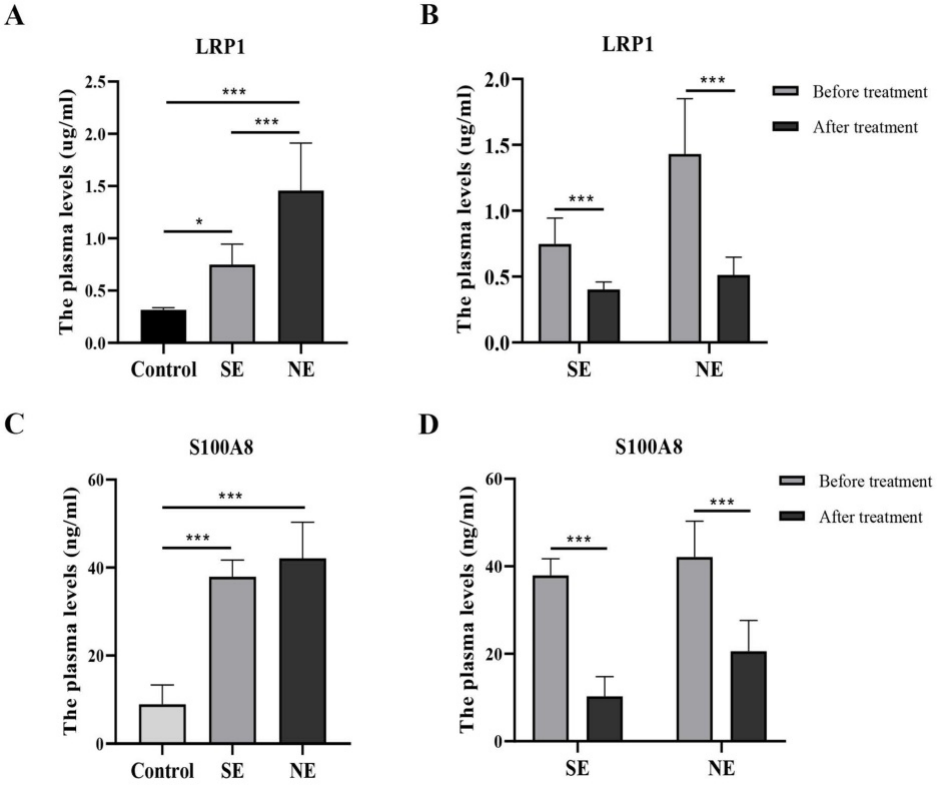
The levels of LRP1 and S100A8 in the plasma of different groups. *P<0.05, ***P<0.001 (SE-the significant effect group, NE-the non-significant effect group, control-non-BPD group).

ELISA analysis showed that LRP1 were significantly different among the three groups before dexamethasone administration (F = 30.42, P < 0.001). Compared with the control group, plasma LRP1 was significantly increased in BPD. Interestingly, the plasma concentration of LRP1 in the NE group was significantly higher than that in the SE group (Fig. 3A). After dexamethasone administration than, the level of LRP1 were differentially downregulated either in the SE group or in the NE group (Fig. 3B). Likewise, S100A8 was significantly different among the three groups before dexamethasone administration (F = 58.61, P < 0.001). The level of S100A8 was significantly higher in both the SE and NE group than that in the control group (P < 0.05). However, there was no significantly difference between the SE and NE group (P = 0.279) before dexamethasone treatment (Fig. 3C). The level of S100A8 was differentially downregulated in both the SE group and NE group after dexamethasone administration than that before dexamethasone administration. (P < 0.05) (Fig. 3D).

## Discussion

In this study, we used DIA technology to analyze the plasma proteins of BPD infants before and after dexamethasone treatment. We found that both LRP1 and S00A8 decreased significantly after dexamethasone treatment. If the baseline LRP1 is high, it suggests that dexamethasone may not show significant effectiveness. This provides a basis for clinical selection of adjuvant corticosteroids to treat BPD.

Dexamethasone had been widely used since the 1980s because of its potent activity against inflammation. The underlined corticosteroid can significantly change the course of BPD and decrease the BPD severity 20. However, the adverse effects (such as cerebral palsy and major neurosensory disability, etc.) caused by glucocorticoids were gradually found from late 1990 21.Therefore, in 2002, the American Academy of Pediatrics had suggested that the use of glucocorticoids in BPD should be more cautious 22. Then the therapeutic use of corticosteroids in BPD had reduced effectively, but the rate of BPD had increased 23. In recent years, glucocorticoid use in BPD infants has been increased. A European study revealed that 13.9% of premature infants (aged less than 30 weeks) received corticosteroid treatment after birth 24, however, in Japan the use of systemic steroid was published by 56% of units in 2005, 57% in 2010, and 68% of units in 2015, respectively 25. There is still no consensus on the use of corticosteroids in BPD 26, 27, 28. In our study, 10 BPD infants were found to be significantly positive response to dexamethasone treatment, and the efficacy of the other 20 patients is not very satisfactory. The results suggested that some sensitive biomarkers are needed to choose the use of adjuvant corticosteroid therapy in BPD.

Protein mass spectrometry had been applied to some experimental studies regarding BPD 29, 30, and clinical research by DIA indicated that calcium-associated proteins can be used as sensitive markers for BPD progress 31. In this study, the results revealed that a total of 52 proteins were differentially expressed before and after dexamethasone treatment. In these differentially expressed proteins, the decreased expression of 43 proteins and elevated expression of 9 proteins were determined. The underlined differentially expressed proteins were further evaluated by literature search and the LRP1 and S100A8 were found to be closely correlated with lung diseases and/or glucocorticoids.

In this study, the plasma LRP1 level in the two BPD subgroups was significantly increased than that in the control group before dexamethasone administration. We also found that, the levels of LRP1 in the SE group were lower than those in the NE group before dexamethasone treatment. These results suggested that LRP1 might be either a diagnostic index or a target of dexamethasone therapy for BPD. These results suggest that a high baseline level of LRP1 may indicate that dexamethasone is not significant effective. This will provide a basis for clinical selection of dexamethasone to treat BPD.

LRP1 was first discovered in 1988, and its amino acid sequence was found to be similar to the LDL receptor. LRP1 is a cell surface receptor and regulates cellular and molecular mechanisms that drive the pathophysiological inflammatory reactions and reorganization of tissues in many organs 32. In current decades, LRP_1_ has been evolved as a significant regulator of the inflammatory reactions. LRP1 negatively regulates adaptive immune responses in the site of HDM-induced eosinophilic airway inflammation 33. LRP has a close association with dexamethasone. The expression of LRP was up-regulated by dexamethasone, and down-regulated by lipopolysaccharide (LPS), gamma interferon (IFN-gamma) or a combination of both. LRP was less sensitive to dexamethasone in activated astrocytes than in microglia 34. Dexamethasone increases cell-surface LRP activity in HepG2 cells by increasing the steady state mRNA levels and suggest that post-transcriptional mechanisms play a role in controlling LRP mRNA levels 35.

Calprotectin S100A8 (S100 calcium binding protein A8, S100A8) is an important member of calcium binding protein family. The evaluation of its role in the pathogenesis of respiratory diseases or its usefulness as a biomarker for the appropriate diagnosis and prognosis of lung diseases have only gained attention in recent years 36. S100A8/A9 is an essential factor for neutrophil recruitment to lungs, and S100A8 promotes acute lung injury *via* Toll-like receptor 4-dependent activation of AECs 37; S100A8/A9 serum levels along with chemokines are useful in distinguishing between active tuberculosis and asymptomatic Mycobacterium tuberculosis-infected latent individuals, and targeting S100A8/A9 pathways as host-directed therapy for TB 38; Calprotectin is significantly elevated in the sputum of Bronchiolitis obliterans patients and reflects ongoing neutrophilic inflammation 39; serum S100A8/S100A9 was negatively associated with pulmonary function in acute exacerbation COPD(AE-COPD) patients, indicating that the serum S100A8/S100A9 heterodimer may be involved in the progression of AE-COPD, and may be a relevant serum biomarker in the diagnosis for AE-COPD 40. Elevated S100A8/S100A9 expression causes glucocorticoid resistance in MLL-rearranged infant acute lymphoblastic leukemia 41. We found that S100A8 levels were significantly different between BPD group and the control group. Unlike LRP1, there was no difference in the level of S100A8 between the SE and NE groups.

In conclusion, we determined some differentially expressed plasma proteins of the dexamethasone significant effect infants with BPD via the DIA technique, among which, LRP1 and S100A8 might be the biomarkers for BPD diagnosis and deciding the use of adjuvant corticosteroids therapy in the BPD infants. This study is only a single center study and the sample number of is still small. In order to support the conclusions of this study, a large sample and multicenter study is necessary.

## Supplementary Material

Supplementary figures.

## Figures and Tables

**Table 1:** Sociodemographic and prerandomization characteristics of study infants [mean ±SD or n (%)]

Perinatal characteristics	SE group (n=10)	NE group (n=20)	X2/t	*P* value
Gestational age (weeks)	31.2 ± 1.1	31.5 ± 1.4	0.092	0.761
Birthweight (grams)	1325.0 ± 249.0	1365.0 ± 262.0	0.053	0.816
Male sex [n (%)]	6 (60)	13 (65)	0.601	0.421
Maternal dexamethasone [n (%)]	9 (90)	18 (90)	—	>0.05
Vaginal delivery [n (%)]	6 (60)	11 (55)	0.093	0.758
Apgar score at 5 min	7 ± 2	7 ± 2	—	>0.05
Surfactant doses administered [n (%)]	10 (100)	20 (100)	—	>0.05
BPD grade (III) [n (%)]	10 (100)	20 (100)	—	>0.05
Respiratory distress syndrome [n (%)]	10 (100)	20 (100)	—	>0.05
Pulmonary hypertension (PH) [n (%)]	2 (20)	5 (25)	0.144	0.739
Patent ductus arteriosus (PDA) [n (%)]	8 (80)	17 (85)	0.629	0.416
Ventilator associated pneumonia [n (%)]	0 (0)	3 (15)	—	<0.01
Atrial septal defect (ASD) [n (%)]	8 (80)	18 (90)	0.838	0.341
Ventricle septal defect (VSD) [n (%)]	8 (80)	17 (85)	1.876	0.170

**Table 2:** Sociodemographic and prerandomization characteristics of dexamethasone sensitive group infants (n = 10)

Perinatal characteristics	DIA group	No-DIA group
n	3	7
Gestational age, weeks	30.0 ± 1.0	30.6 ± 1.5
Birthweight, grams	1350 ± 160	1365 ± 180
Male sex, n (%)	2 (66.7)	4 (57.1)
Maternal dexamethasone, n (%)	3 (100.0)	6 (85.7)
Vaginal delivery, n (%)	2 (66.7)	4 (57.1)
Apgar score at 5 min	7 ± 2	7 ± 2
Respiratory distress syndrome, n (%)	3 (100.0)	7 (100.0)
Surfactant doses administered, n (%)	3 (100.0)	7 (100.0)
BPD grade (III), n (%)	3 (100.0)	7 (100.0)

There were no significant differences between groups. Data are mean ± SD or n (%).

**Table 3:** The information table of significant difference protein between after and before treatment of corticosteroids (top 10 proteins)

id	Symbol	log2fc	*P* value
ENSG00000123384	LRP1	-2.764152705	0.006
ENSG00000127083	OMD	-1.713222868	0.013
ENSG00000129538	RNASE1	-1.75694677	0.019
ENSG00000143546	S100A8	-2.012564987	0.043
ENSG00000011052	NME	-13.43760929	0.0459
ENSG00000167815	PRDX2	-1.75665693	0.165
ENSG00000090013	BLVRB	-2.106522502	0.183
ENSG00000285043	AC093512.2	-2.21657055	0.211
ENSG00000111341	MGP	-2.787895194	0.222
ENSG00000041982	TNC	-2.69720062	0.366
