# Supplementary material for: A clinical study on plasma biomarkers for deciding the use of adjuvant corticosteroid therapy in bronchopulmonary dysplasia of premature infants
Source: Int J Med Sci. 2021 Apr 29;18(12):2581–8. doi: 10.7150/ijms.58650 (PMC8176188; doi:10.7150/ijms.58650)
Supplement: Supplementary file 1 — Supplementary figures. [file ijmsv18p2581s1.pdf]

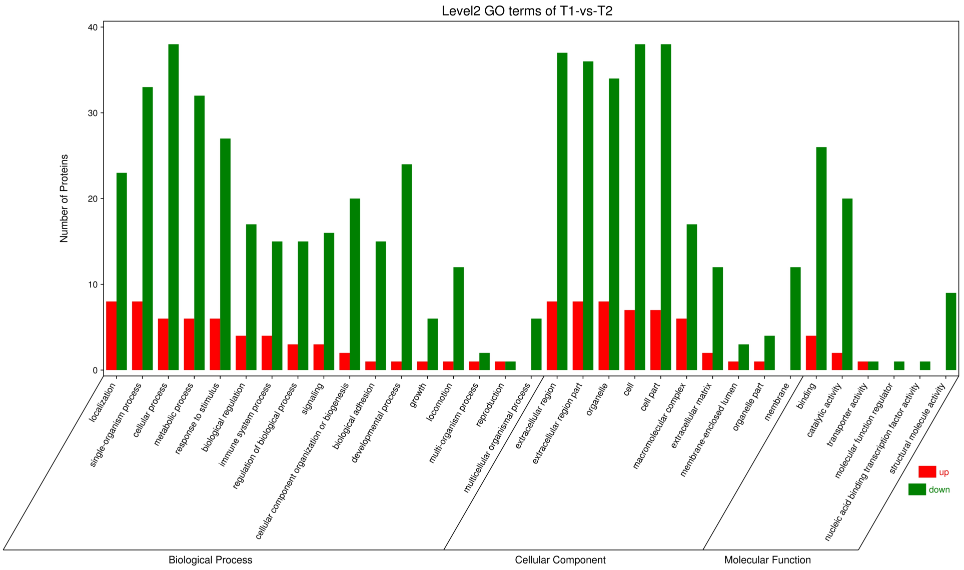

Supplementary Online Fig.1 Gene ontology (GO) analysis of differentially expressed proteins.

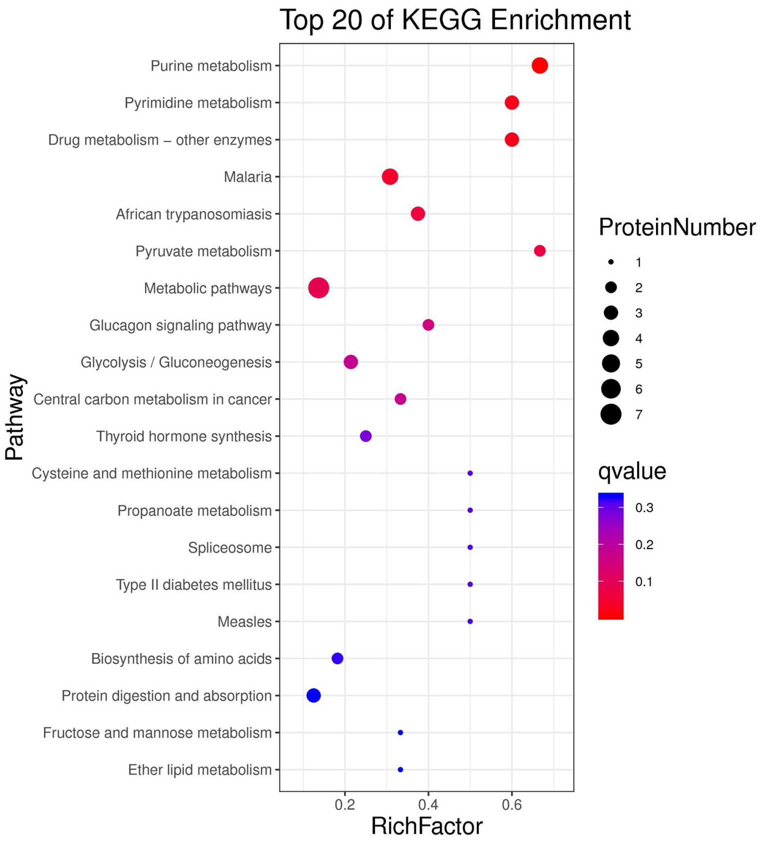

Supplementary Online Fig.2. KEGG analysis of differentially expressed proteins

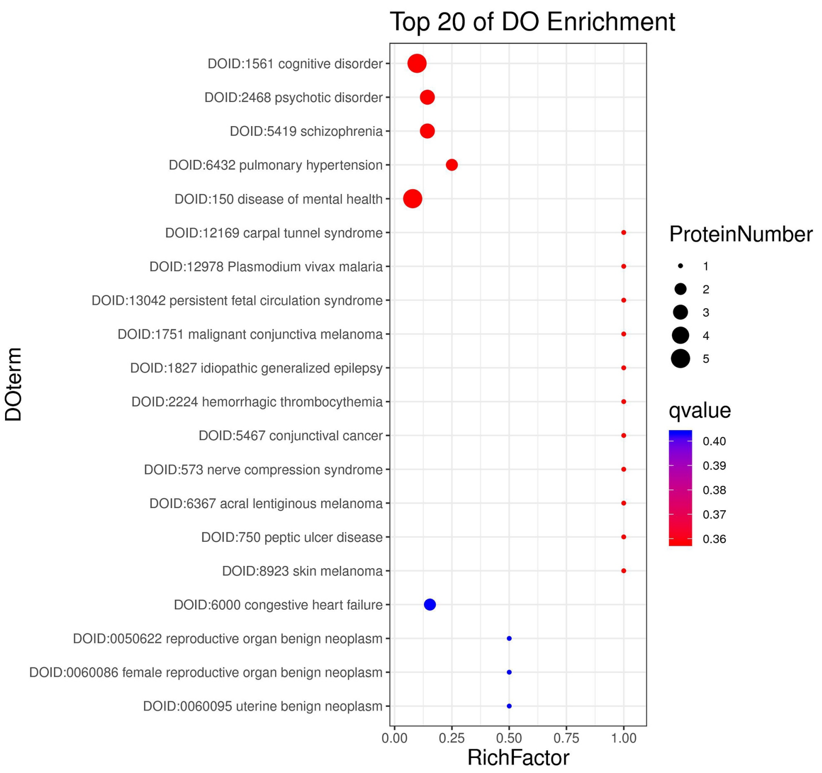

Supplementary Online Fig.3. Disease Ontology (DO) analysis of differentially expressed proteins.

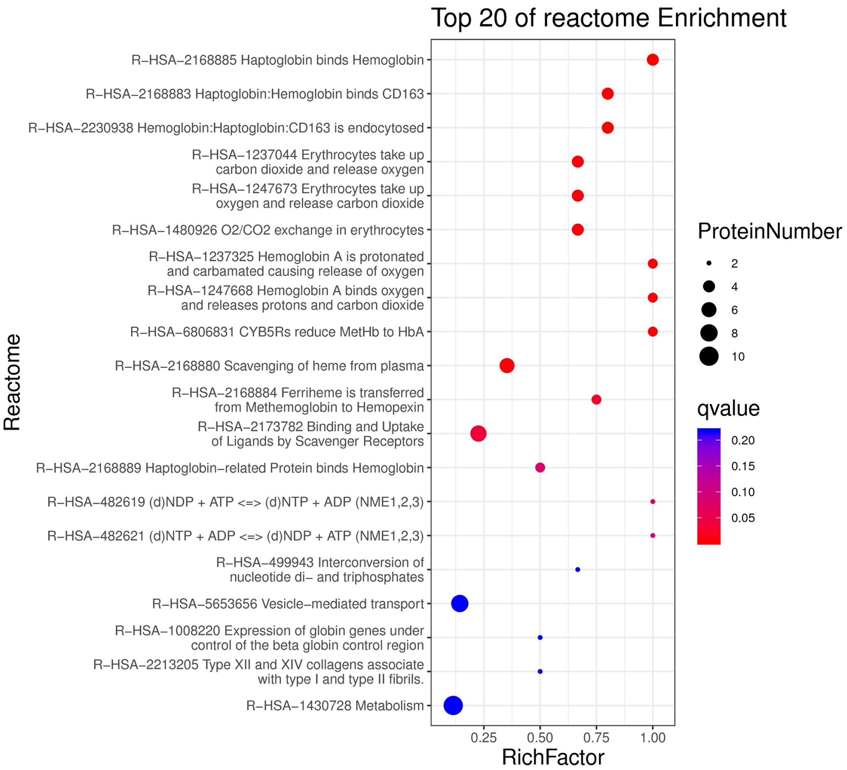

Supplementary Online Fig.4 Reactome enrichment analysis of differentially expressed proteins.
